# Endophytic Fungi: From Symbiosis to Secondary Metabolite Communications or Vice Versa?

**DOI:** 10.3389/fpls.2021.791033

**Published:** 2021-12-17

**Authors:** Beena Alam, Jùnwén Lǐ, Qún Gě, Mueen Alam Khan, Jǔwǔ Gōng, Shahid Mehmood, Yǒulù Yuán, Wànkuí Gǒng

**Affiliations:** ^1^State Key Laboratory of Cotton Biology, Key Laboratory of Biological and Genetic Breeding of Cotton, The Ministry of Agriculture, Institute of Cotton Research, Chinese Academy of Agricultural Sciences, Anyang, China; ^2^Department of Plant Breeding & Genetics, University College of Agriculture and Environmental Sciences, The Islamia University of Bahawalpur (IUB), Bahawalpur, Pakistan; ^3^Biotechnology Research Institute (BRI), Chinese Academy of Agricultural Sciences, Beijing, China

**Keywords:** symbiosis, endophytic fungi, endophytic fungi-host interaction, genetic regulation, secondary metabolites

## Abstract

Endophytic fungi (EF) are a group of fascinating host-associated fungal communities that colonize the intercellular or intracellular spaces of host tissues, providing beneficial effects to their hosts while gaining advantages. In recent decades, accumulated research on endophytic fungi has revealed their biodiversity, wide-ranging ecological distribution, and multidimensional interactions with host plants and other microbiomes in the symbiotic continuum. In this review, we highlight the role of secondary metabolites (SMs) as effectors in these multidimensional interactions, and the biosynthesis of SMs in symbiosis *via* complex gene expression regulation mechanisms in the symbiotic continuum and *via* the mimicry or alteration of phytochemical production in host plants. Alternative biological applications of SMs in modern medicine, agriculture, and industry and their major classes are also discussed. This review recapitulates an introduction to the research background, progress, and prospects of endophytic biology, and discusses problems and substantive challenges that need further study.

## Introduction

Plants, especially perennials, are colonized by many types of endophytic microorganisms (Stone et al., 2004; Demain, 2014), which live inside plant tissues either throughout their lives or during a certain period of their life cycles without causing visible damage or morphological changes in their hosts. These microorganisms include both fungi and bacteria (Zhang et al., 2006; Porras-Alfaro and Bayman, 2011; Gouda et al., 2016), and usually coexist with pathogens. According to their colonizing behaviors, endophytic microflora can be sorted into facultative and obligate categories. Facultative endophytes colonize plants at certain stages of their life cycles, but they may also reside outside the plant at other stages to form an association with the immediate rhizosphere soil of host plants (Abreu-Tarazi et al., 2010). In contrast, obligate strains live in plants throughout their entire life cycles. They usually proliferate across plant generations through vertical transmission and use or alter the metabolic machinery and products of plants for their own survival (Hardoim et al., 2008; Gouda et al., 2016).

Among these endophytic microorganisms, endophytic fungi (EFs) have attracted much research interest because they have provided not only novel sources of cytotoxic compounds, such as anticarcinogenic molecules (Uzma et al., 2018) and antibacterial substances (Radic and Strukelj, 2012), but also biostimulants for essential oil biosynthesis (El Enshasy et al., 2019). They may enhance nutrient solubilization in the plant rhizosphere (Mehta et al., 2019), promote plant growth (Poveda et al., 2021), act as biological control agents (Poveda and Baptista, 2021), or activate plant systemic resistances to biotic (Poveda et al., 2020a) or abiotic (Cui et al., 2021) stresses. In this review, we provide a comprehensive overview of the biological aspects of EFs that are related to their diversity, their distribution, and their multidimensional interactions with multiple players, including host plants, epiphytes, and pathogens, in their communities. We also examine the role of secondary metabolites (SMs) in these multidimensional interactions and how SMs are biosynthesized through gene expression regulation and through the mimicry or alteration of phytochemical production in host plants. Finally, we identify the principal categories of SMs, characterize their attributes, and review their alternative biological potential. This review provides readers with a profound understanding of EFs and SMs.

## Biodiversity and Distribution of Endophytic Fungi in Nature

The geographical distribution of biota is characterized as a continuous gradient distribution of traits. Biodiversities at various levels, including species, function, and phylogenesis, are the basis of this continuous distribution (Violle et al., 2014). Although little is known about the mechanisms of biodiversity formation in a particular geographical habitat, fungal strains mediate many processes and may play a crucial role in their habitats (Vandenkoornhuyse et al., 2002). Plant tissue is arranged in multi-layers, forming a spatial and temporal supportive refuge, like a natural habitat, for various endophytic microorganisms. Based on an accepted estimation of a 1:4 or 1:5 ratio of vascular plants to fungal strains, there could be more than one million strains of EFs remaining to be discovered (Sun and Guo, 2012). However, our limited recognition of EF diversity renders the ratio a biased estimation, because EFs thrive ubiquitously in species diversity, while rare species and those that are characterized as non-sporulating, non-culturable, or asceptic cannot be examined properly in current laboratory isolation and fermentation attempts (Stone et al., 2004; Zhang et al., 2006; Ding et al., 2017; Alvear-Daza et al., 2021).

According to the reproductive pattern and host occurrence, EF communities can be sorted into two categories: the Clavicipitaceous/Balansiaceous group (C-group) and the non-Clavicipitaceous/non-Balansiaceous group (NC-group). C-group EFs infect the ovules of host plants and transmit vertically from parents to progenies through host seeds. The target tissues for their colonization are living rhizomes and shoots of host plants, but the host range is restricted to grass species (*Poaceae*). C-group species are typical obligate endophytes (Carroll, 1988), which protect their hosts from herbivore attacks or enable the hosts to survive under drought conditions by secreting defensive or supportive bioactive metabolites, respectively (Roberts and Lindow, 2014; Poveda, 2021). NC-group EFs, which are non-grass-host related (*Ascomycota*, *Basidiomycota*), have a wide biodiversity and distribution from tropical to polar areas, with their hosts including nonvascular, vascular, and woody plant communities. They transmit sexually or asexually by producing spores or conidia, which contribute to horizontal propagation, i.e., the induction of symbiosis. These EFs are not closely associated the host plants because they can exist in a quiescent state until they sense the chemical changes from host plants suffering injuries, wounds, or other environmental stresses (Carroll, 1988; Rodriguez et al., 2008; Mishra et al., 2021). The colonization of the NC-group in aerial organs is usually local, restricted, limited, and mainly intercellular, but the colonization of this group in roots or the rhizosphere is extensive, organized, systematic, intercellular, and intracellular. Some illustrative endophytic mycobiomes of the NC-group include *Fusarium* spp., *Piriformospora indica*, and dark septate mycobiota (Varma et al., 2000; Schulz et al., 2002).

Recent progress in molecular techniques, such as metagenomic sequencing, DNA fingerprinting, and phylogenetic analysis, has been successfully employed to detect and identify the species, community composition, and diversity of EFs. These technologies provide more precise methods of fungal identification and accommodation of the asceptic strains *in situ* than conventional isolation attempts (Arnold and Lutzoni, 2007; Tao et al., 2008; Tejesvi et al., 2009; Bahram et al., 2018; Jiang et al., 2018; Huang et al., 2019; Zhang J. et al., 2019). Studies have demonstrated that the host range of EFs includes algae (Tan and Zou, 2001), liverworts, mosses, hornworts (Davey and Currah, 2006; Aly et al., 2010; Sun and Guo, 2012), grasses (Muller and Krauss, 2005; Su et al., 2010), lycophytes, ferns, equisetopsida (Aly et al., 2010), shrubs, deciduous and coniferous trees (Gao et al., 2008; Albrectsen et al., 2010; Mohamed et al., 2010; Sun et al., 2011), gymnosperms, angiosperms, and annual/perennial herbaceous and broad-leaved plants. EFs are also distributed in a broad range of geographic habitats, such as tropical, temperate, arctic tundra, alpine, aquatic, and xerophytic ecosystems (Tan and Zou, 2001; Stone et al., 2004; Zhang et al., 2006) for more than 400 million years (Sun and Guo, 2012).

## Endophytic Fungi and Their Multidimensional Interactions

**FIGURE 1:**
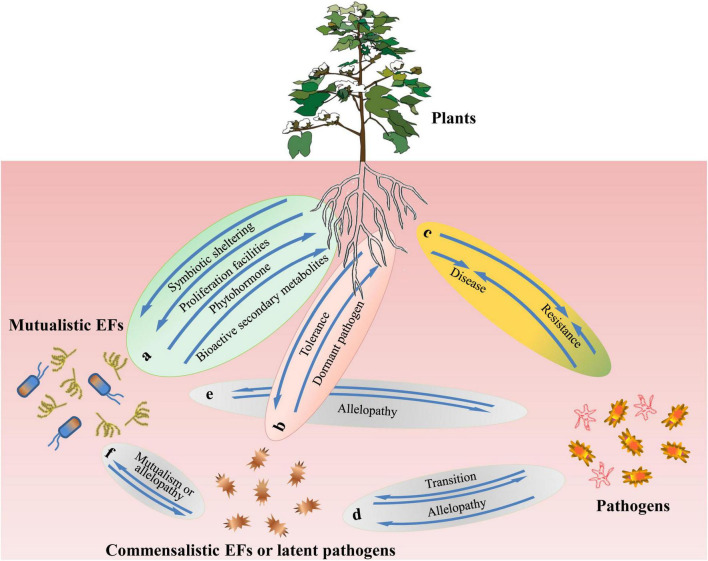
A schematic model of plant and microbiome interactions. The interactions include: **(a)** interactions between plants and mutualistic endophytic fungi (EFs), **(b)** interactions between plants and commensalistic EFs or latent pathogens, **(c)** interactions between Plants and pathogens, and **(d–f)** interactions between microbiomes. 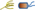 Mutualistic endophytic fungus (EF),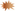 commensalistic 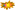 EF, 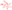 pathogens.

During their biogenesis and the establishment of symbiosis, EFs encounter specific host groups (Stone et al., 2004; Sun et al., 2008; Chadha et al., 2014), non-host plant communities, epiphytes, and pathogens. These multiple encounters prompt EFs to develop multidimensional interactions with the organisms they encounter (Figure 1). Major evolutionary and ecological novelties may direct or correlate to these interactions.

### Endophytic Fungi Interactions With Host Plants

Fungi can colonize the intercellular or intracellular spaces of plants, but systematic and extensive colonization is most likely to occur in the roots rather than in the aerial leaves or stems. Colonization in aerial organs primarily depends on the host’s apoplastic fluid as the nutrient source to support the normal reproduction of EFs in aerial organs (Schulz et al., 2002). During infection, fungi form three corresponding types of interactions with hosts: mutualistic (beneficial endophytes), commensalistic (non-beneficial/virulent endophytes), and pathogenic (virulent pathogens) (Figure 1), depending on the physiological status or specific circumstances that host plants experience. According to these three modes of action, fungal strains can increase, have no palpable effects on, or decrease host fitness (Kogel et al., 2006).

#### Mutualistic Symbiosis

In mutualistic symbiosis, both partners of EFs and host plants enjoy this beneficial symbiotic continuum (Jia et al., 2016) and eventually achieve evolutionary and ecological success. EFs alter the metabolic mechanism of host plants, improve metal and drought tolerance, enhance growth, and promote nutrient acquisition (Hunt and Newman, 2005; Rodriguez and Redman, 2008; Mejia et al., 2014; Poveda et al., 2021). They also enhance the defense efficiency of host plants against herbivorous animals and pests as well as against infections of pathogenic microorganisms (Cui et al., 2021). In response to these benefits, host plants provide symbiotic shelters and other proliferation facilities, such as an adequate nutrient supply and protection, to enable fungi to safely complete their life cycles during colonization (Figure 1a). The exact mechanism of mutual interaction between EFs and plants remains to be elucidated (Haas and Défago, 2005), but EFs offer these potential advantages to host plants in several possible ways. One of these ways is to enhance the plant’s immune system by producing a plethora of bioactive SMs as protective agents. It is speculated that increasing the number of SMs causes physiological changes in the infected host plant that further stimulate the plant immune system (Poveda et al., 2020a). Various experiments on endophytic and pathogenic fungi and monitoring their comparative effects on host plants suggest that EFs promote the defense mechanisms of host plants by synthesizing bioactive SMs and herbicidal metabolites in hosts (Figueiredo et al., 2008). In an *in vitro* tripartite interaction assay, it was observed that *Fusarium oxysporum* rapidly killed *A. thaliana* plants, whereas the presence of *Paraconiothyrium variabile* reduced plant death by up to 85% (Bärenstrauch et al., 2020). This hypothesis was confirmed in the following experiments. When mosquito larva were assayed with ethanol extracts from *Poa ampla* Merr. (big bluegrass), the results showed that only the extracts from the plants that were inoculated with *Neotyphodium typhnium* were effective against the insect, whereas the extracts from the plants that were not inoculated with the fungus were inactive (Ju et al., 1998). Another way that an EF bestows host plant benefits is that EFs promote plant growth by producing and providing phytohormones, including auxins, cytokines, and gibberellins. This has been confirmed by the discovery of a common gibberellin biosynthesis pathway in fungi and higher plants (Tan and Zou, 2001; Kumar et al., 2013). Studies have demonstrated that endophytes can improve the growth and proliferation of plants by enhancing their defensive systems, like ethylene and jasmonic acid do (Kunkel and Brooks, 2002; Van der Ent et al., 2009; Di et al., 2016; Verbon and Liberman, 2016; Forni et al., 2017; Yang et al., 2019), or by interacting with ethylene-targeted transcription factors (Camehl et al., 2010). *Neotyphodium*, an EF, colonizes in tall fescue ryegrass and confers protection and stability on host plants in hostile conditions; in return, ryegrass provides facilities to promote fungal proliferation through ryegrass seeds infected with fungal hyphae (Tan and Zou, 2001). The resulting competitive advantage provides both hosts and symbionts with greater potential for growth and survival than the non-symbiotic plants and fungi.

#### Commensalistic to Latent Pathogenic Relationships

In a commensalistic or latent pathogenic relationship, EFs sporulate rapidly and interact with host plants through a relationship with or without any significant beneficial effects on plant physiology (Stone et al., 2004; Hiruma et al., 2016). Studies have reported that these endophytes exist as latent pathogens in hosts under normal conditions (Brown et al., 1998; Photita et al., 2004; Rodriguez and Redman, 2008; Gorzynska et al., 2018; Figure 1b), while some studies have described various species and genera of EFs from host plants as active pathogens under unusual physiological stresses (Photita et al., 2004; Figure 1c). Fungi that have been identified as endophytes that are also possible pathogens include *Cladosporium*, *Fusarium*, *Colletotrichum*, *Cordana*, *Deightoniella*, *Periconiella*, *Verticillium*, *Curvularia*, *Nigrospora*, *Guignardia*, and *Phoma* (Photita et al., 2004; Cui et al., 2021). These EFs stay in latent or dormant state in the tissue of their host plants long before the outbreak of disease symptoms. In such cases, the dormancy phase is essential because it determines the time when the fungus is harmless as an endophyte and when it is virulent as a pathogen. In the virulent phase, EFs cause obvious symptoms and change the morphology and physiology of host plants under adverse conditions (Figure 1d). It is precisely these hostile conditions, including malnutrition, disruption of ontogenetic state (Sieber, 2007; Rodriguez and Redman, 2008), biotic stresses, drastic climate changes (such as elevated temperature and excessive humidity), and senescence, that break the balance between EFs and their hosts and lead to the transition of EFs from latent mode to active virulent pathogens, although there are no obvious disease symptoms before transition (Romero et al., 2001; Photita et al., 2004; Poveda et al., 2020b). There are also endemic fungal species, which typically include the majority of *F*. *oxysporum* strains, that live in host tissues without causing disease symptoms. Some strains even confer beneficial effects (Imazaki and Kadota, 2015; Di et al., 2016), such as *C*. *tofieldiae*, which promotes plant growth and fertility as an endophyte under phosphorus-deficient conditions (Hiruma et al., 2016). It is assumed that a combination of effectors, enzymes, and secondary metabolites determines the outcome of an interaction; that is, whether it is endophytic or pathogenic (Di et al., 2016; Poveda et al., 2020b). Nutrient status may have facilitated the transition of *C*. *tofieldiae* from pathogenicity to symbiosis (Hiruma et al., 2016).

### Endophytic Fungi Interactions With Other Plant Microbiomes

As one of the numerous microbial players in the endophyte–host continuum, EFs inevitably have dynamic and complex interactions with other plant microbial communities, including endosphere-associated fungal and bacterial strains, regardless of whether they are pathogenic or symbiotic under natural conditions (Strobel, 2018; Figures 1d–f). Studies have observed that fungal endophytic metabolomic profiles can be affected by pathogen infection (Combes et al., 2012), indicating that antagonistic effects, or chemical communications, exist between the two microorganisms (Combes et al., 2012; Saikkonen et al., 2013). Endophytic fungi may also harbor a variety of bacterial species (endohyphal bacteria) in their hyphae (Hoffman and Arnold, 2010). When a foliar endophyte hosts the endohyphal bacterium *Luteibacter* sp., its indole acetic acid (IAA) production is significantly enhanced. However, the axenic culture of the bacterium does not show IAA production (Hoffman et al., 2013). Host plants may provide direct interfaces facilitating interactions between EFs and bacterial microflora. It is observed that two mutualistic EFs, *Neotyphodium* sp. and *Epichloë* sp., protect the leaves of fescue grasses from herbivores by producing loline alkaloids. On the leaf surface of fescue grass that is not infected with such endophytes or on the leaf surface of other plants that are infected with endophytes incapable of producing loline alkaloids, there is no accumulation of loline-consuming bacteria (Roberts and Lindow, 2014). In some cases, indirect interactions between EFs and other microorganisms may involve the participation of a third organism. A typical example is the interaction between the EF *N*. *coenophialum* and grass yellow dwarf virus (Hunt and Newman, 2005). *N*. *coenophialum* protects its host plants from aphids, and aphids are primary vectors of viruses (Grafton et al., 1982; Malmstrom et al., 2005; Yi and Gray, 2020). The *N*. *coenophialum* strains that provide tall fescue with better prevention against bird-cherry-oat aphids also inhibit the spread of the virus (Hunt and Newman, 2005).

## Why Do Endophytic Fungi Produce Secondary Metabolites?

**FIGURE 2:**
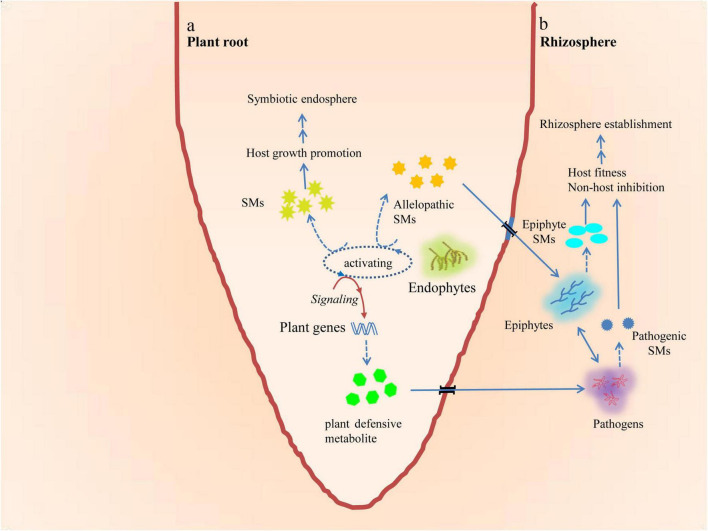
Mutual orientation of microbiome and plant **(a)** and multidirectional crosstalks or chemical communications among plants, endophytes, epiphytes, and pathogens under natural ecological conditions **(b)**. 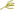 Endophytic fungus (EF), 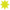 EF secondary metabolite (SM), 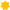 allelopathic SMs, 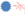 pathogens, pathogenic SM, 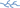 epiphytes, 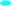 epiphytic SM, 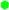 plant defensive metabolite.

When different microorganisms occupy the same habitat, they must compete for the resources of that habitat for nutrition, living space, reproduction, and other needs throughout their life cycles. Compared to microorganisms that have poor adaptability, adaptable microorganisms are more likely to obtain adequate resources and increase their abundance when the resources are insufficient to meet the needs of the community. As a result, the former may not be able to survive in adverse conditions. In order to survive, organisms have developed two effective strategies to compete. One is to produce allelochemicals that inhibit the growth of their competitors and eliminate toxic effects produced by their competitors in the vicinity (Macías-Rubalcava et al., 2008; Konarzewska et al., 2020; Poveda, 2021). The other is to produce allelochemicals that help their producers form alliances through symbiotic relationships with symbionts or hosts. These symbiotic relationships enable both parties to survive and reproduce safely, even in extremely adverse environments (Macías-Rubalcava et al., 2008). According to the hypothesis of long-term coevolution within biological communities (Ji et al., 2009), this mutual orientation of EFs and their hosts leads to each EF having developed a specific range of host species, enabling them to accumulate in a specific eukaryotic host group (Figure 2a).

Most of these allelochemicals are SMs, a variety of usually low-molecular-weight and amazingly heterogeneous chemicals that were previously thought to have no direct functional effect on the growth, development, and reproduction of the organisms that produce them (Keller et al., 2005; Yu and Keller, 2005; Fox and Howlett, 2008; Shwab and Keller, 2008). Volatile organic compounds (VOCs) are a large group of such chemicals that allow their producers (including plants and microorganisms) to defend themselves against attacks by pests or pathogens, or to convey warnings intra- or inter-specifically during such attacks (for details of VOCs, please refer to Poveda, 2021). Historically, the term “secondary” used for such natural metabolites has been associated with their “inessentiality,” but they have been demonstrated to play important roles in the growth and development of organisms in indirect ways (Pichersky and Gang, 2000; Deepika et al., 2016).

### Secondary Metabolites Serve as Agents to Help Endophytic Fungi Compete and Survive

By preventing competition from other organisms, SMs help the organisms that produce them survive. They may also cause harm to other individuals of the same species. Examples of these SMs include those that help organisms access limited resources and survive in a specific adverse niche and those that inhibit competitors (Williams et al., 1989; Vaishnav and Demain, 2010; Xie et al., 2019; Figure 2b). In an *in vitro* competition experiment, naphthoquinone spiroketals, isolated from a newly identified EF strain, exhibited an allelochemical inhibitory effect against other EFs, such as *Colletotrichum* sp., *Phomopsis* sp. (Wang et al., 2016), and *G*. *manguifera* (Macías-Rubalcava et al., 2008; Macías-Rubalcava et al., 2014). In another *in vitro* dual culture experiment, it was observed that *P. variabile*, an EF, exhibited direct antagonism against *F. oxysporum*, a phytopathogen, by secreting the induced metabolite hydroperoxin oxylipin, which decreased the concentration of pathogenic mycotoxins, whereas none of the pure axenic cultures showed an increase or decrease of this metabolite (Combes et al., 2012). Further study has revealed that the biosynthesis of hydroperoxy oxylipins is catalyzed by lipoxygenases and the two lipoxygenase genes (*pvlox1* and *pvlox2*) in *P. variabile*, and that only *pvlox2* is specifically up-regulated during the interaction (Bärenstrauch et al., 2020).

### Secondary Metabolites Form the Phylogenesis of Symbiosis of Endophytic Fungi and Their Host Plants

The concept of coevolution has been applied to characterize the biochemical interactions between EFs and hosts. It is thought that the coevolution of endophytes and their host plants shapes the production of SMs, which play important roles in endophyte-host communication for mutual adaptation and their orientation to different environments (Debbab et al., 2011; Lind et al., 2017). There are three main schools of thought to interpret the relationship between the biosynthesis pathway of common SMs and the evolution of symbiosis between endophytes and their hosts. According to the hypothesis of the first school, this may occur in the era of coevolution when a responsive relationship has been established between EFs and host plants. EFs and host plants were adapted to each other, leading them to share common biosynthetic pathways of natural active SMs. In this case, the endophyte–host association is imperative because it may be the critical factor affecting the secretion of bioactive metabolites (Vaishnav and Demain, 2010). The environmental factors that affect symbiosis formation will also affect SM biosynthesis. The second school of thought suggests that endophytes gradually acquired adaptations to the internal microenvironment of hosts by horizontal gene transfer (HGT) between endophytes and plants during the primeval period (Slot and Rokas, 2011; Soanes and Richards, 2014). In such genetic variations, fungal strains insert some fragments of their genetic materials into plant genomes, or uptake some plant genetic fragments into their own genomes (see section “Horizontal Gene Transfer”). The third school of thought has argued that both endophytes and plants synthesize these common metabolites and transfer them to their corresponding symbiotic systems (Zhang et al., 2006).

Some investigations support the hypothesis that phytoendophytes originate from phytopathogens. Phylogenetic analysis has revealed the interconnections between phytoendophytes and phytopathogens in various environments (Arnold et al., 2009). SMs might also be involved in such interconnections (Di et al., 2016). Recent studies reported that the transient evolutionary roots of two fungal endophytic communities, Clavicipitaceous sp. *N*. *coenophialum*, an endophyte of tall fescue, and *Harpophora oryzae*, a beneficial endosymbiont of wild rice, originated from insect parasitoid and phytopathogenic ancestors, respectively (Spatafora et al., 2007; Xu et al., 2014). The exact phylogenetic mechanism of endophytes from apparent pathogens still remains unclear. It has been observed that the transfer of one specific *F. oxysporum* f. sp. *lycopersici* chromosome, which contains most of its effector genes and a secondary metabolite coding gene cluster, confers pathogenicity to an endophytic strain (Ma et al., 2010; Di et al., 2016). Studies suggest that environmental stresses, sudden drastic climate changes, or senescence may facilitate the conversion of endophytes into pathogens in the host to adapt to these ecological changes (Romero et al., 2001; Photita et al., 2004). They may also play a role in phylogenesis from pathogens to endophytes (Cui et al., 2021). Given such a prolonged virulent contradiction between pathogens and plants, the loss-of-function mutations of the virulence genes in pathogenic strains and the alteration of the SM biosynthetic pathway and accumulation of SMs eventually convert such pathogen–host interactions into mutual symbiotic relationships that are beneficial to both parties (Tan and Zou, 2001; Kemen et al., 2015). It has been observed that plants infected with EFs have more bioactive chemical compounds than those infected with pathogenic fungi (Schulz et al., 2002). Generally, when facultative EFs begin to colonize host plants, they face three kinds of competitors: epiphytes, pathogens, and the host defense systems. This may explain why plants infected with EFs produce more defensive chemicals than plants infected with pathogens (Schulz et al., 2002; Hunt and Newman, 2005; Figure 2b). These examples illustrate the significance of metabolic communications for the construction of multiple interactions among EFs, host plants, and other plant microbiomes, and for the identification of diverse metabolic chemicals secreted by plants or EFs during their interaction (Friesen et al., 2011; Quambusch et al., 2014; Hardoim et al., 2015).

### Diverse Secondary Metabolites Help Form Endophytic Fungi Diversity and Thus Enhance Plant Diversity

In competitive and unfavorable ecological environments, plant species modify their biological systems by producing various defense reactions, which are mainly manifested in the synthesis of defensive SMs. It is estimated that plant genomes may contain more than 70,000 genes (Wang et al., 2018), of which 15–25% are involved in encoding enzymes that participate in secondary metabolism (Bevan et al., 1998; Somerville and Somerville, 1999). This indicates that defensive SMs are often highly diverse within and across populations. EFs also take part in the establishment of host defense mechanisms through SMs to enhance the host capacity to adapt to a wide range of biogeographical ecosystems (Violle et al., 2014). One reason for the diversity of the SMs of an EF species is the diverse arrangement of coding genes in the SM synthetic pathway. As the coding genes of SM synthetic pathways in EFs are usually in clusters (Andersen et al., 2013), the divergent rearrangements of the clusters within an EF species or across EF species may also contribute to the diversity of SMs. Recently, at least five divergent types of variation in SM gene clusters in the fungal species *Aspergillus fumigatus* were identified, revealing its diverse secondary metabolism (Lind et al., 2017). Although their mechanism is still poorly understood, SMs may cause the morphological or physiological alteration of host plants (Braga et al., 2018). These alterations are believed to be linked to plant diversity, which means that the colonization of EFs enhances the host adaptability and thus its viability under various environmental conditions (Hyde and Soytong, 2008); in return, the survival of EFs may also be enhanced through their host diversifications.

## How Do Endophytic Fungi in Symbiosis Produce Diverse Secondary Metabolites?

### Gene Expression Regulation of Secondary Metabolite Biosynthesis in Endophytic Fungi

Analyzing the gene expression regulation mechanism of EFs, their hosts, and their symbiosis contributes greatly to further understanding the interactions between the two symbiotic partners (Deepika et al., 2016). The expression of SM coding genes in a symbiotic continuum may be regulated by factors including gene clustering, transcription factors, the presence of EFs, and HGT.

#### Gene Clustering

The diversity of EF genomes in eukaryotic systems and fungal genetic studies has revealed that genes coding specific SM biosynthesis pathways are clustered. In some cases, such as when the pathway function requires specific transcription factors or transporters, the genes encoding these factors (enzymes) are also located in these clusters (Nützmann et al., 2018). Two findings suggest that the architecture or structure of fungal secondary metabolic clusters varies with the situation. First, some highly complex SMs are synthesized through collaboration between different clusters (Chiang et al., 2016; Henke et al., 2016). Second, in some cases clusters of different pathways are adjacent to each other in the genome (Nützmann et al., 2018). These clusters are usually found in the dynamic regions of chromosomes or near telomeres. Subtelomeric regions are well-known hotspots in chromosomal recombinations and segmental duplications (Field et al., 2011; Andersen et al., 2013; Lind et al., 2017). The mechanism of SM-coding gene clusters in unstable DNA regions is still unclear, but an acceptable explanation is that it may be related to gene expression regulation (Osbourn, 2010a). The proximity of clustered genes may be a necessary condition for the synthesis of bioactive products related to a pathway, because it keeps the pathway genes closer during genomic rearrangements (Deepika et al., 2016). This clustering genetic format may facilitate the co-inheritance of favorable combinations of alleles at these multigene loci (Chu et al., 2011; Field et al., 2011). It may also monitor the synchronization of the clustered gene expression by altering the arrangement of chromatins (Hurst et al., 2004; Sproul et al., 2005; Field and Osbourn, 2008; Osbourn and Field, 2009; Osbourn, 2010b; Field et al., 2011) and by exchanging the non-contiguous regulatory elements, which are harbored in the clusters (Hurst et al., 2004; Sproul et al., 2005; Osbourn and Field, 2009; Osbourn, 2010b). In the genomes of higher plants, the functionally correlated genes interspersed in the genome may form clusters at the transcriptional level through helix-loop-helix domains (Nützmann et al., 2018). The formation of DNA loops causes cis-elements to be located adjacent to each other and creates high local concentrations of transcription factors that are close to the transcription initiation sites of the genes, thereby initiating transcription (Mendes et al., 2013). Any interference with the transcription of clustered genes will not only cause the loss of the coding products of these genes, but may also cause toxic intermediates to accumulate in the biochemical pathways (Hurst et al., 2004; Sproul et al., 2005). Evidence indicates that gene regulation at chromatin levels is important for the expression of secondary metabolic gene clusters (Osbourn, 2010b).

#### Transcription Factors

The activities of these clustered genes in the secondary metabolism are further regulated by two main groups of transcription factors: narrow domain transcription factors (NDTFs) (Table 1) and broad domain transcription factors (BDTFs) (Table 2; Keller et al., 2005). NDTFs act on the genes in the cluster, and may also act on the clustered genes at different genomic locations from NDTFs themselves. This can be illustrated by the typical NDTF AflR, which is a well-characterized Zn_2_Cys_6_ transcription factor that regulates the aflatoxin and sterigmatocystin gene clusters through binding to the palindromic sequence 5′-TCG(N5)CGA-3′, an 11-bp motif in the promoter regions of coding genes in a few *Aspergillus* species (Slot and Rokas, 2011). It also regulates three more genes outside the aflatoxin metabolite gene cluster (Woloshuk et al., 1994; Yu et al., 1996; Cánovas et al., 2017). Some typical NDTFs are shown in Table 1. BDTFs, or global transcription factors, are upper hierarchical-level control systems that respond to external stimuli that are not directly related to secondary biochemical gene clusters (Keller et al., 2005; Yu and Keller, 2005; Young et al., 2006; Hoffmeister and Keller, 2007; Fox and Howlett, 2008; Shwab and Keller, 2008; Deepika et al., 2016). Studies have revealed that EF signals interfere preferentially with ethylene-targeted transcription factors (Camehl et al., 2010). It is well-accepted that the biosynthesis of SMs depends on a combination of developmental competence and the stimulation of environmental factors, such as nutrient availability, illumination, pH, injury, infection, and developmental changes during the host life cycle (Bayram and Braus, 2012; Xie et al., 2019). BDTFs play an essential role in the transmission of environmental stimuli to the genome. They create and regulate the signaling transduction from environmental cues to cellular responses in the formation of specific SMs (Keller et al., 2005). Some typical BDTFs are shown in Table 2.

**TABLE 1:** Narrow domain transcription factors (NDTFs).

Transcriptional factors (regulatory proteins)	Class of regulatory transcriptional proteins	Microfungal organisms	Functional metabolites	References
AflR	Zinc binuclear cluster protein of Zn_2_Cys_6_ type	*A. flavus* and *A. parasiticus*	Aflatoxin and sterigmato cystin	Woloshuk et al., 1994; Chang et al., 1995; Yu et al., 1996; Fernandes et al., 1998; Slot and Rokas, 2011
AflJ	Zn_2_Cys_6_ DNA-binding protein	*A. parasiticus*	Aflatoxin and sterigmato cystin	Meyers et al., 1998; Chang et al., 2000
MlcR	Zinc binuclear cluster protein of Zn_2_Cys_6_ type	*P. citrinum*	Compactin	Abe et al., 2002; Deepika et al., 2016
ApdR	GAL4-type Zn_2_Cys_6_ need to check	*A. nidulans*, *A. flavus*	Aspyridone A and B	Bergmann et al., 2007
GliZ53	Zinc finger transcription factor GliZ53	*A. nidulans*	Gliotoxin	Cramer et al., 2006
PENR1	HAP-like transcription factor	*A. nidulans*	Penicillin and some enzymes like cellobiohydrolase, xylanase, and taka-amylase	Brakhage et al., 1999
ToxE	Ankyrin repeat protein	*Cochliobolus* sp.	HC-Toxin	Pedley and Walton, 2001; Keller et al., 2005
AcFKH1	2-Peptide forkhead protein	*A. chrysogenum*	Cephalosporin C	Schmitt et al., 2004
CPCR1	2-Peptide forkhead protein	*A. chrysogenum*	Cephalosporin C	Schmitt et al., 2004
Tri4, Tri5, Tri6	Cys_2_His_2_ zinc finger proteins	*F. sporotrichioides*	Trichothecene	Proctor et al., 1995
MRTR14, MRTR15, MRTR16	Cys_2_His_2_ zinc finger proteins	*F. sporotrichioides*, *Myrothecium roridum*	Trichothecene	Trapp et al., 1998; Keller et al., 2005; Liu et al., 2016

**TABLE 2:** Broad domain transcription factors (BDTFs)/global transcription factors.

Transcriptional factors (regulatory proteins)	Class of regulatory transcriptional proteins	External cues	Microfungal strains	Functional metabolites	References
CreA	Zinc finger protein of Cys_2_His_2_ type	Carbon signaling	*A*. *nidulans*	Penicillium	Shwab and Keller, 2008; Deepika et al., 2016
PacC/CBC	Zinc finger protein of Cys_2_His_2_ type	Alkaline pH signaling	*A*. *nidulans*	Penicillium/ β-lactam	Shwab and Keller, 2008; Deepika et al., 2016
AreA	Zinc finger protein of Cys_2_His_2_ type	Nitrogen signaling	*F*. *fujikusori*	Gibberellins	Deepika et al., 2016
AreA	Zinc finger protein of Cys_2_His_2_ type	Nitrogen signaling	*F*. *verticillioides*	Fumonisin B1	Kim and Woloshuk, 2008
FadA/homologous of FadA	G-protein signaling regulator	Growth related hormone like extracellular ligands	*A. nidulans*/ *F. sporotrichioides*	Penicillium/ trichothecene	Tag et al., 2000
PkaA	Protein kinases	Growth related cues jointly work with G-proteins	*A. nidulans*	Penicillium	Hicks et al., 1997; Shimizu and Keller, 2001
FlbA	G-protein signaling regulator	Asexual sporulation cues	*A. nidulans*	Aflatoxin and sterigmatocystin synthesis	Deepika et al., 2016
HapB, HapC, HapE, HapX	CCAAT-binding complex	pH, Iron-depriving, and redox status signaling	*A. nidulans*	Penicillium, iron-scavenging siderophores	
LaeA, VeA, VelB	Velvet complex	Light dependent regulatory developmental cues	*A. nidulans*, *A. chryroseum*, *P. chryroseum*, *A. flavus*	β-lactam, aflatoxin	Kato et al., 2003; Dreyer et al., 2007; Bayram et al., 2008; Kosalkova et al., 2009; Bayram and Braus, 2012
LaeA	Protein methyltransferase	Light dependent signaling	*A. nidulans*, *A. fumigatus*, *A. terreus*	Sterigmatocystin (ST) biosynthesis, chromatin modification	Bok and Keller, 2004; Lee et al., 2005

**FIGURE 3:**
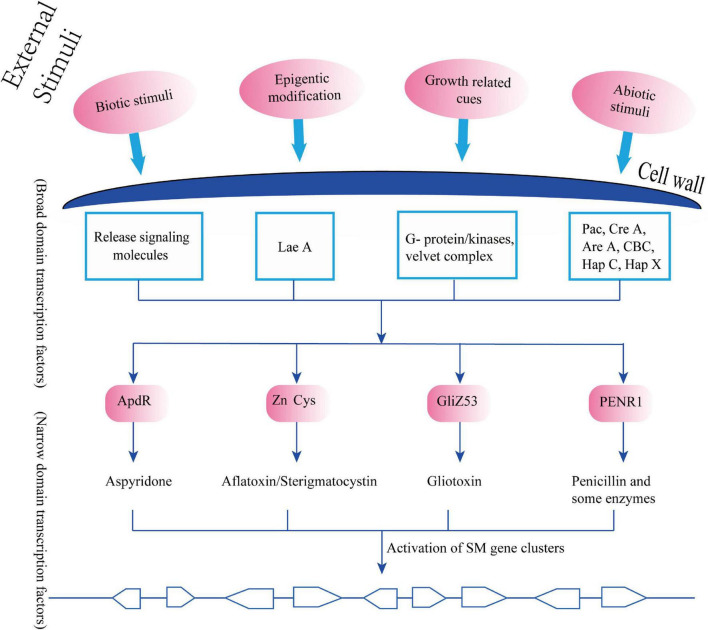
Synchronized regulatory model of secondary metabolites (SMs) biosynthesis and external/morphological indicators.

The close relationship between NDTFs and BDTFs has been extensively reviewed by various reports, which have explained how BDTFs or global transcription factors perceive the environmental and developmental cues and transduct these external messages to NDTFs through chromatin and histone modification or through specific biochemical cascade reactions, including methylation, phosphorylation, and acetylation. These reactions are essential for activating the silent clustered genes associated with specific SMs that are required in particular cellular metabolisms, growth stages, or environmental conditions (Figure 3; Foss et al., 1993; Kouzminova and Selker, 2001; Tamaru and Selker, 2001; Calvo et al., 2002; Yu and Keller, 2005; Shwab and Keller, 2008).

#### Alterations in Plant Genetic Makeup in the Presence of Endophytic Fungi

Plant phenotypes depend not only on the genetic makeup of the plant, but also on the activity of microbiome and environmental factors (Jia et al., 2016). It has been observed that the presence of EFs enhances the resistance potential of host plants to biotic and abiotic stresses (Rodriguez et al., 2008; Shoresh et al., 2010; Busby et al., 2017; Yan et al., 2019) or improves nutrient uptake (Xie et al., 2019). Although the exact mechanism still remains to be elucidated, evidence has shown that plant genetic expression profiles are altered in the presence of EFs (Mejia et al., 2014; Cui et al., 2017; Liao et al., 2019; Xie et al., 2019). Endophytes reprogram the host genomic expression through epigenetic interactions with the host. In epigenetic events, DNA methylation and demethylation induced by endophytes enhance the host’s defensive processes (Kouzminova and Selker, 2001; Strobel, 2018; Geng et al., 2019). EFs stimulate the immune systems of plants and increase the quantity of SMs (Cui et al., 2017; Xie et al., 2019), which may induce some physiological changes in infected host plants. Bailey et al. (2006) regarded these altered or differentiated gene expressions in the presence of EFs as a complex system of genetic crosstalk between EFs and hosts. During the crosstalk, the genomic expressions of both endophytes and hosts are altered. Additional examples are given in Table 3.

**TABLE 3:** Selected examples of plant gene alteration in the presence of endophytic fungi (EFs) and their resulted beneficial expression.

Host Plant	Endophytic fungal community	Gene ontology (up regulation/down regulation)	Phenotypic expression/gene function	References
Taxus (young/old plantlets)	*Paraconiothyrium* SSM001	Up regulation of TS, DXR, HMGR genes (related to taxol synthesis)	Increase the concentration of host plant taxol	Soliman et al., 2013
Soybean plant (*Glycine max*)	*P. indica*	61 genes are up- regulation and 238 genes are down-regulated	Enhancing the iron transport, lignin biosynthesis, hormone signaling, nutrient acquisition, biosynthesis of phenylpropanoids, flavonols, siderophores, and flavonoids (61 genes) 238 genes involved in encoding the heat shock protein, and several other abiotic stress related defense responses	Bajaj et al., 2018
*Rhodiola crenulata*	*Trimmatostroma* sp. ZPRs-R11	Up regulatory genes are TYDC, MAOA, C4H, TAT, PAL, PCD	Induced the accumulation of tyrosol and salidroside	Cui et al., 2017
*Lolium perenne* (perennial ryegrass)	*N*. *lolii*	Up regulation of MRP/PDR like ABC and GST transporter systems related genes and downregulation of carbohydrate metabolism and photosynthesis related genes	Activates the cellular transport, transporter system, protein synthesis.	Khan et al., 2010
*Hordeum vulgare* L. (barley)	*P. indica*	PR-5 gene	Induced tolerance in salt stress and the systemic disease resistance *via* elevating the conc. of antioxidant metabolites (ascorbate-glutathione pathway).	Waller et al., 2005

#### Horizontal Gene Transfer

Horizontal gene transfer refers to the exchange of genetic materials between isolated lineages through asexual processes. A growing body of evidence has suggested that frequent HGT occurs between independent organisms and between EFs and their hosts (Richards et al., 2011; Bapteste et al., 2012). Most HGTs identified in microbiota are associated with invading, degrading, and manipulating hosts (Soanes and Richards, 2014), which supports the hypothesis that HGT may be a robust source of novel adaptive traits of EFs and that HGT is a strong driving force for fungal adaptive evolution (Feurtey and Stukenbrock, 2018). As the coding genes of a metabolic pathway are always located in a cluster, HGT of an intact metabolic cluster may enable the recipient organism to obtain a new and complete metabolic pathway (Slot and Rokas, 2011). Bapteste et al. (2012) identified a particular evolutionary unit of genetic materials that introgress into different host structures and propagate within these structures. These evolutionary units may leave recognizable patterns in resemblance networks. This finding suggests that HGT also plays an important role in the emergence of new fungal strains (Feurtey and Stukenbrock, 2018).

The expression of horizontally transferred genes in recipient organisms is the first step for HGT to play a role, and determine the colonization mode and capacity of EFs in host plants (Soanes and Richards, 2014). The mechanism of how horizontally transferred genes initiate their expression in recipient organisms remains to be discussed. Slot and Rokas (2011) demonstrated that when the gene cluster encoding the biosynthesis of sterigmatocystin (a toxic SM) was transferred from an *Aspergillus* species to *Podospora anserine*, the transferred cluster was functional because it was expressed in the latter. Many studies have demonstrated that EFs can produce host-mediated compounds, whereas the latter can also produce some EF-originated metabolites. For instance, djalonensone has been detected as a fungal metabolite in various *Alternaria* fungi, but it was first identified in extracts from cauliflower plants. Similarly, the EF metabolite aureonitol produced by *Chaetomium* sp. can also be detected in the extract of *Helichrysum aureonitens*. Many reported EF-originated compounds, such as alternariol, alternariol monomethyl ether, altenusin, macrosporin, and methylalaternin, were further detected in their host plants, *Polygonum senegalense* and *Urospermum picroides*. These results provide evidence for HGT or genetic recombination during the coevolution between hosts and endophytes, thus forming the genetic regulation mechanism of SM biosynthesis (Aly et al., 2010; Kozyrovska, 2013).

When HGT occurs between prokaryotes and eukaryotes, the integrated genes are more likely to be expressed in a modified manner in the recipient organism due to the fundamentally distinct gene expression mechanisms between the two genomes. The failure of HGT expression may lead to the appearance of pseudogenes in recipient organisms (Andersson, 2009).

### Endophytic Fungi Mimic or Alter Phytochemical Production in Host Plants

The wide range of biotic and abiotic stresses that plants constantly face in natural or agricultural environments lead to specific transcriptional variations at the individual gene level, with a high degree of variability and stress specificity (Zhang and Sonnewald, 2017). This was supported by a study of plant responses to combined heat and drought stress, in which the genes involved in secondary metabolism were significantly up-regulated (Prasch and Sonnewald, 2013). In symbiosis, EFs mimic the production of SMs in hosts using primary phytocompounds as precursors. In contrast, proteins secreted by EFs may potentially act as effectors altering host metabolism (Spanu et al., 2010; Soanes and Richards, 2014; Kemen et al., 2015), with the specificity of each effector targeting a distinct hormone signaling pathway (Di et al., 2016).

#### Endophytic Fungi Sense the Same Stimuli as Hosts

A “xenohormesis” hypothesis proposed by Howitz and Sinclair (2008) suggests that heterotrophic organisms (animals and microbes) may sense plant signals or molecules induced under stress. By doing so, the heterotrophic organisms may take advantage of the defensive responses of autotrophs to increase their own survival chances. EFs may also have the ability to sense chemical cues in plants, and begin to produce similar SMs (Kusari et al., 2012). As ecological adaptions and survival elements, SMs are not synthesized throughout the whole life cycle, but only when necessary, especially in the early differentiation or late senescence stages of an organism (Deepika et al., 2016; Stahl et al., 2018; Siddhardha and Meena, 2020). In general, their synthesis is minimal when the organism grows rapidly and is maximal when the growth of the organism ceases. For an organism, the core indicator point of its synthesis of SMs is that it is in a state of rapid response rather than in a state of rapid growth (Deepika et al., 2016). In such circumstances, the genetic materials of both EFs and their hosts are cross-activated under hostile conditions (Stierle et al., 1993; Zhou et al., 2010). The best example, which well supports the previous hypothesis, is the pioneering identification of the anti-carcinogenic drug Taxol (paclitaxel), which is a worldwide selling cancer drug, with annual sales of million USD, from an EF, *Taxomyces andreanae*. This EF was originally identified from *Taxus brevifolia*, the host plant of *T. andreanae*, indicating that both the EF and its host produce the same SM in response to environmental stimuli. Since its initial discovery in the past few decades, Taxol has been proven to be more efficiently produced in the EF than in the host. Studies also show that Taxol can be effectively isolated from several other EF strains and Pacific yew plants (Stierle et al., 1993; Zhou et al., 2010; Chandra, 2012; Zaiyou et al., 2017; Uzma et al., 2018).

#### Endophytic Fungi Share Common Precursors With Hosts

Primary metabolites are the end products of primary metabolic pathways, including carbohydrates, amino acids, proteins, and lipids. They play a primary metabolic role in the construction and development of an organism. Without them, the growth and development of the organism are at extreme risk for defects. An important role of primary metabolism is that the products of some key steps provide precursors for the synthesis of SMs. Endophytes and their host plants share these precursors in their respective SM biosynthesis pathways. The biosynthetic pathway of SMs in EFs may be the result of their mimicking of the host pathways (Kirby and Keasling, 2009; Deepika et al., 2016). The synthetic pathways of some phytochemicals, including ergot alkaloids, aflatoxin, and lovastatin, have been studied by blocking mutant and radio labeling techniques (Keller et al., 2005). It has been revealed that although diverse, SMs are produced by a few common biosynthetic pathways and the metabolomic pathways of endophytic fungal communities and their host plants are similar. At this stage, the question still remains of whether these low molecular weight phytochemicals are synthesized by plants or as a result of symbiosis with microorganisms living inside their tissues. The combination of some possible inducers has promoted the accumulation of bioactive metabolites in EFs and plants, indicating that EFs play a significant role in the biosynthesis of SMs. These inducers include nutrient deficiencies, morphological development, and growth rates (Kirby and Keasling, 2009; Deepika et al., 2016).

## Classes of Fungal Secondary Metabolites and Their Biological Potential

**FIGURE 4:**
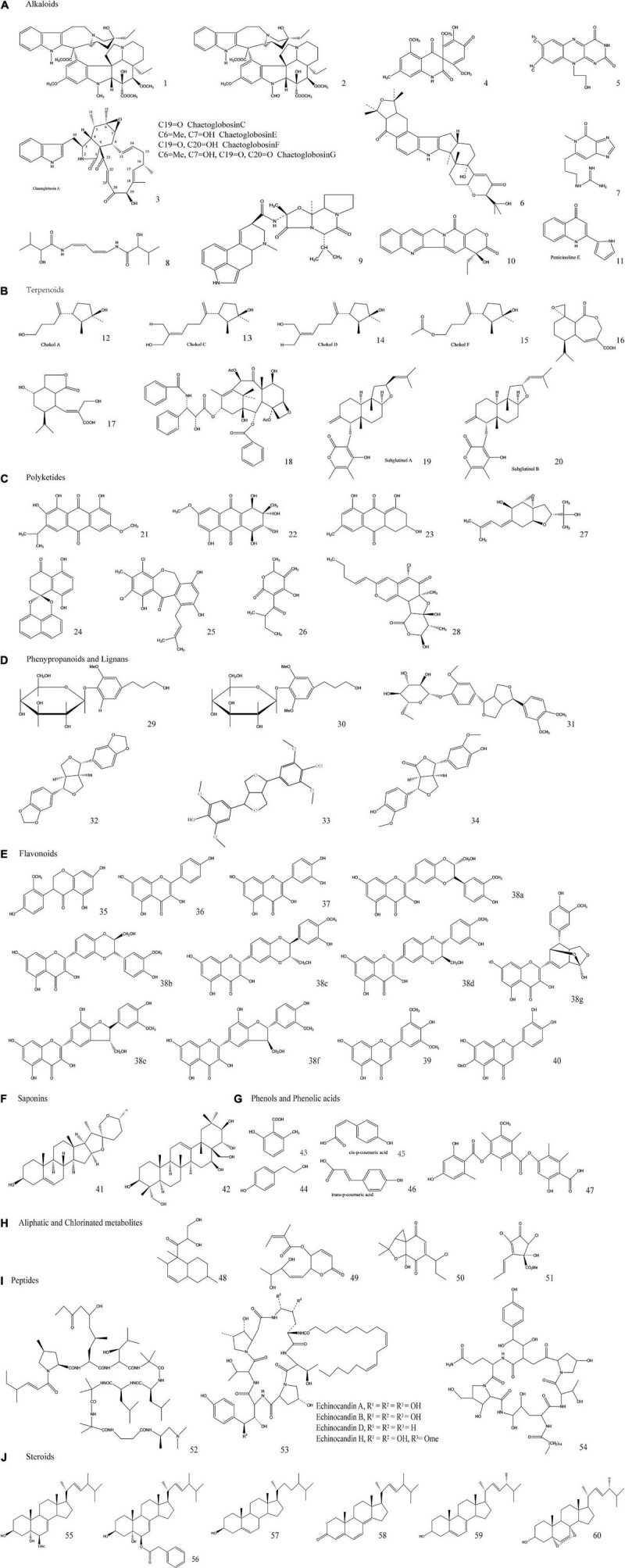
Molecular structures of some typical secondary metabolites (SMs). The classes, subclasses, compounds with references, endophytic fungal sources, together with their potential biological properties of the SMs are briefed in Table 5.

EFs are considered to be rich sources of diverse bioactive SMs and phytohormones to support plant growth and enable plants to survive under biotic or abiotic stresses (Tan and Zou, 2001; Chadha et al., 2014). Some of the most important commercially exploited SMs, including antibiotics, anticarcinogenics, cytotoxics, insecticides, and allelopathic compounds, can also be biosynthesized by EFs (Schneider et al., 2008). They have broad, promising commercial prospects in the pharmaceutical, medical, agricultural, nutraceutical, cosmetic, flavor, and fragrance industries, making EFs an attractive topic in the field of endophytism research (Hyde and Soytong, 2008; Table 4). Therefore, exploring the benign symbiotic relationship that synthesizes these bioactive SMs between EFs and plants and its impact on the genetic materials of EFs and plants will provide a promising framework for the discovery and development of new bioactive SMs through metabolomic and genetic engineering in the future (Kirby and Keasling, 2009; Deepika et al., 2016). Of the numerous SMs biosynthesized by EFs and hosts, most can be sorted into the following classes: alkaloids, terpenoids, polyketides, phenylpropanoids and lignins, flavonoids, saponins, phenols and phenolic acids, aliphatic, and chlorinated metabolites, peptides, and steroids. To facilitate the comparison of these SMs, they are presented in tabular form in Table 5 and Figure 4 after a brief description of their EF sources, chemical structures, and biological application potential.

**TABLE 4:** Commercial applications of bioactive natural products with endophytic fungi (EFs)-based biogenesis.

Application fields	Reported products	Endophytic fungal sources	References
Pharmaceuticals	Taxol (anticarcinogenic agent)	*Paraconiothyrium* SSM001	Soliman et al., 2013
	Cycloepoxytriol B (antibiotic agent)	*Phomopsis* sp.	Hussain et al., 2009
Flavor and fragrance	Methyl eugenol [1,2-dimethoxy 4-(2-propenyl) benzene]	*Alternaria* sp.	Kaul et al., 2008
Cosmetics (cream, shampoos, lotions, toothpaste, etc.)	Fatty acids (e.g., oleic, stearic, linoleic, and palmitic acid)	*Bionectria ochroleuca*, *C*. *truncatum*, *Chaetomium* sp.	George et al., 2011; Kumar and Kaushik, 2013; Yang Y. et al., 2015
	Chitosan	*A. flavus*, *C. cladosporioides, Phoma* sp.	George et al., 2011
Food industry	Chitosan (as food additive)	*A. flavus*, *C. cladosporioides, Phoma* sp.	Liu et al., 2008; George et al., 2011
	7-amino-4-methylcoumarin (food preservative agent)	*Xylaria* sp.	Liu et al., 2008
Bioinsecticides	Loline alkaloids	*N*. *uncinatum*	Aly et al., 2010
Bioherbicides	Ascotoxin (growth inhibitory effect)	*Paraconiothyrium* sp.	Khan et al., 2012
Nutraceuticals	Saponins	*Aspergillus*, *Bulgaria*, *Penicillium*, *Phomopsis* sp.	Nicoletti and Fiorentino, 2015

**TABLE 5:** Classes of endophytic fungal secondary metabolites (SMs) with biological potential activities.

Classes of SMs	Sub classes of SMs	Compounds with references	Endophytic fungal sources	Chemical structures*	Potential biological properties
a. Alkaloids	Indole derivative alkaloids	Vinblastine, vincristine (Keglevich et al., 2012; Kumar et al., 2013)	*F. oxysporum*	1, 2	Antitumor drugs
		Chaetoglobosin (Zhang Y. et al., 2012; Huang et al., 2016)	*C. elatum*	3	Antitumor activity against breast tumor and cholangiocarcinoma cell lines
	Pyridines and pyrrolizidines	Asperfumoid (Zhang Y. et al., 2012; Li et al., 2015)	*Penicillium* sp.	4	Potent cytotoxic
		7,8-dimethyl-isoalloxazine (Owen and West, 1971; Zhang Y. et al., 2012; Li et al., 2015)	*Penicillium* sp.	5	Cytotoxic agent
		Lolines (Bush et al., 1997; Tan and Zou, 2001)		6	Allelopathic and insecticidal properties
	Amines and amides	Peramine (Schardl and Phillips, 1997; Tan and Zou, 2001)	*Neotyphodium* sp., *Epichloë* sp.	7	Insecticidal- pyrrolopyrazine alkaloid
		Phomoenamide (Rukachaisirikul et al., 2008)	*Phomopsis* sp.	8	Antibacterial properties
		Ergovaline (Flieger et al., 1997; Duringer et al., 2007; Rukachaisirikul et al., 2008; Zhang Y. et al., 2012)	*Neotyphodium* sp., *Claviceps* sp.	9	Neurotoxicity in livestock (feeding repellent)
	Quinoline and isoquinoline	Camptothecin (Sriram et al., 2005; Shao et al., 2010; Zhang Y. et al., 2012; Wu et al., 2015)	*Nothapodytes fortida*	10	Potent cytotoxic drug, antiprotozoal, and anti-HIV properties
		Penicinoline and its derivatives (Shao et al., 2010; Zhang Y. et al., 2012; Bladt et al., 2013; Naveen et al., 2017)	*Penicillium* sp., *Auxarthron reticulatum*, and mangroves associated endophytic fungal species	11	Cytotoxic compound
b. Terpenoids	Sesquiterpenes	Chokols and its derivatives (A, C, D, F) (Hiroyuki et al., 1989)	*E. typhina*	12, 13, 14, 15	Fungicidal properties against *C. phlei* pathogen
		Heptelidic acid and hydroheptelidic acid (Calhoun et al., 1992; Tan and Zou, 2001)	*Phyllosticta* sp.	16, 17	Toxic against *C. fumiferana* larvae
	Diterpenes	Taxol (paclitaxel) (Nicolaou et al., 1994; Tan and Zou, 2001; Lin et al., 2014)	*T. andreanae*	18	Anticarcinogenic drug
		Subglutinol A and B (Nicolaou et al., 1994; Tan and Zou, 2001; Lin et al., 2014)	*F. subglutinans*	19, 20	Immunosuppressive property
c. Polyketides		6-O-Methylalaternin (Mousa and Raizada, 2013)	*Ampelomyces* sp.	21	Biocontrol agent against parasitic fungi
		Altersolanol A (Mousa and Raizada, 2013)	*A. solani*	22	Antibiotic (antibacterial) properties
		Palmarumycin CP17 (Martínez-Luis et al., 2008)	*Edenia* sp. (*Pleosporaceae*)	23	Antiparasitic compound especially against protozoans, antineoplastic effects *via* G_2_/M stage in mammalian cell cycle
		Rugulosin (Mousa and Raizada, 2013)	*Hormonema dematioides*	24	Act as a mycotoxins due to having cell necrosis, fatty acids degeneration effects makes it a natural cytotoxic compound
		Pestalachloride B (Li et al., 2008)	*P. adusta*	25	Antibiotic (antifungal) activities
		CR377 (Brady and Clardy, 2000; Mousa and Raizada, 2013)	*Fusarium* sp.	26	Antibiotic (antifungal) activities
		Pestalotheol C (Mousa and Raizada, 2013)	*Pestalotiopsis theae*	27	Inhibitory effect
		Chaetomugilin A (Qin et al., 2009)	*C. globosum*	28	Cytotoxic effect against brine shrimp larvae
d. Phenylpropanoids and lignans		Coniferin (Falshaw et al., 1969; Chapela et al., 1991; Daubresse et al., 1997)	*Xylariaceae* sp.	29	Reduced the biosynthesis of lignins *via* inhibition of oxidases
		Syringin (Eleutheroside B) (Falshaw et al., 1969; Chapela et al., 1991; Cho et al., 2001; Li et al., 2017)	*Xylariaceae* sp.	30	Antioxidant effects, anti-inflammatory, immunomodulatory, and most remarkably used in cardiac disease (cardiac hypertrophy)
		Phillyrin (Zhang Q. et al., 2012; Chen et al., 2016)	*C*. *gloeosporioides*	31	Antioxidant, anti-inflammatory, and antipyretic activities
		Sesamin (Lee et al., 2011; Nicoletti and Fiorentino, 2015)	*A. ilanense*	32	Antitumor, antioxidantive, antihypertensive properties
		Syringaresinol (Cheng et al., 2013; Nicoletti and Fiorentino, 2015; Kim et al., 2016)	*A. ilanense*	33	Activating the SIRT1 gene expression, leading to slow the cellular senescence, and enhanced the function of endothelial cells
		4-Ketopinoresinol (Chen et al., 2012; Cheng et al., 2013; Nicoletti and Fiorentino, 2015)	*A. ilanense*	34	Nrf2/ARE-mediated transcription activator and eliminate the oxidative stress effects
e. Flavonoids		Cajanol (Liang et al., 2013; Zhao et al., 2013; Nicoletti and Fiorentino, 2015)	*Hypocrea lixii*	35	Anticarcinogenic and antimalarial properties
		Kaempferol (Vellosa et al., 2011; Nicoletti and Fiorentino, 2015)	*F. chlamydosporum*	36	Cytotoxic and antioxidant properties
		Quercetin (Materska, 2008; Huang et al., 2013; Nicoletti and Fiorentino, 2015)	*A. ilanense*	37	Reduce degenerative disease, apoptotic activity against liver cancer, antioxidant drug
		Silymarin (AbouZid, 2012; Nicoletti and Fiorentino, 2015)	*A. iizukae*	38a–38g including 7 flavonolignans (silybin A, B, isosilybin A, B, silychristin A, B, and silydianin)	Anti-inflammatory, anticarcinogenic, anti-asthma, hyperprolactinemia, hepatoprotective, immunostimulant
		Tricin (Tan and Zou, 2001; Mousa and Raizada, 2013)	*N. typhnium* infected bluegrass	39	Toxic effect against mosquito larvae and acted as antimalarial agent
		Flavones glycosides (Tan and Zou, 2001; Mousa and Raizada, 2013)	*N. typhnium* infected bluegrass	40	Antimalarial agent
f. Saponins		Diosgenin (Nicoletti and Fiorentino, 2015)	*Fusarium* sp., *Cephalosporium* sp., *Paecilomyces* sp.	41	Pharmaceutically effective drug and important precursor of progesterone, corticosteroids, and other several steroidal drugs
		Gymnemagenin (Nicoletti and Fiorentino, 2015)	*P. oxalicum*	42	Antidiabetic properties
g. Phenols and phenolic acids		2-Hydroxy-6-methyl benzoic acid (Yang et al., 1994; Zou et al., 2000)	*Phoma s*p.	43	Antibiotic activity
		Tyrosol (Zou et al., 2000; Rodríguez-Morató et al., 2015)	*E. typhina*	44	Antifungal
		*cis*- and *trans*- *p*-coumaric acids (Zou et al., 2000; Sigurdson et al., 2018)	*E. typhina*	45, 46	Antimicrobial activities
		Colletotric acid (Zou et al., 2000)	*C*. *gloeosporioides*	47	Antimicrobial compound
h. Aliphatic and chlorinated metabolites		Phomodiol (Calhoun et al., 1992; Horn et al., 1996)	*Phomopsis* sp.	48	Antimicrobial, insecticidal, algicidal properties
		Phomopsolide B (aliphatic ester related compounds) (Tan and Zou, 2001)	*Phomopsis* sp.	49	Antimicrobial activities
		Mycorrhizin A (Tan and Zou, 2001)	*Phyllosticta* sp. strain 76	50	Antibiotic drug
		Cryptosporiopsin (chlorinated compounds) (Tan and Zou, 2001)	*Pezicula* sp., *P. livida*	51	Algicidal drug
i. Peptides		Leucinostatin A (Tan and Zou, 2001)	*Acremonium* sp.	52	Fungicidal, antitumor, phytotoxic properties
		Echinocandins A, B, D, H (Tan and Zou, 2001)	*A. rugulosus*, *Cryptosporiopsis* sp., *Pezicula* sp.	53	Antibiotic activities
		Cryptocandin (Tan and Zou, 2001)	*Cryptosporiopsis cf*. *quercina*	54	Antifungal properties
j. Steroids		3β,5α-dihydroxy-6β-acetoxyergosta-7,22-diene and 3β,5α-dihydroxy-6β-phenylacetoxyergosta-7,22-diene (Lu et al., 2000)	*Colletotricum* sp.	55, 56	Fungicidal activity
		3β-hydroxyergosta-5-ene and 3-oxoergosta-4,6,8(14),22-tetraene (Lu et al., 2000)	*Colletotricum* sp.	57, 58	Fungicidal activity
		Ergosterol (Yu et al., 2010; Yang H. et al., 2015; Nowak et al., 2016)	*Nodulisporium* sp.	59	Antimicrobial activity
		5a, 8a-epidioxy ergosterol (Yu et al., 2010; Nowak et al., 2016)	*Nodulisporium* sp.	60	Antimicrobial activity

*The chemical structure of the secondary metabolite represented by each number is shown in Figure 4.

**a. Alkaloids**

Alkaloids include indole derivatives, pyrimidine and pyrrolizidine, quinoline and isoquinoline, amines, and amides (Zhang Y. et al., 2012). They are isolated from EFs that colonize in grass (Clavicipitaceous genera: *Epichloë* sp.) (Guerre, 2015), and because of their toxicity, they enable hosts to inhibit insect and herbivore attacks. In addition, their concentration levels are mainly dependent on EF strains, species, or genotypes and less dependent on environmental factors and host genotypes (Zhang et al., 2006; Gundel et al., 2018). Alkaloids are composed of bioactive compounds. In addition to complex chemical properties, alkaloids have bioactive properties, such as fungicidal, antibiotic, and antitumor activities, making them the main focus of numerous drug research and development projects (Zhang et al., 2006; Song et al., 2020).

**b. Terpenoids**

Terpenoids are the second major group of plant SMs. They also are obtained from endophytic mycobiota. Their primary characteristics include defensive agents, pollinator attractors (mainly odoriferous or color components), and allelochemicals in competitive environments. Among terpenoids, sesquiterpenoids, and diterpenoids can be easily isolated from endophytic cultures without degradation (Jakubczyk and Dussart, 2020).

**c. Polyketides**

Polyketides are the most copious and well-characterized fungal metabolites, and are exclusively detected in mycospecies. Polyketide synthases play the most important role in their biosynthesis. These synthases are similar to those of eukaryotic fatty acid, but their products are different due to the selective reduction of the β-carbon in polyketides rather than the compulsive reduction of that position in fatty acids (Keller et al., 2005; Jakubczyk and Dussart, 2020).

**d. Phenylpropanoids and lignins**

These types of SMs are produced in wounds and lesion conditions to protect the host from pathogenic attacks. Lignin compounds play a role in initiating wound healing (Yadav et al., 2020). EFs also produce these phytochemicals to improve the adaptability of host plants to adversity. These compounds can be isolated from both infected plants and separately cultured EFs (Dixon and Paiva, 1995; Sampangi-Ramaiah et al., 2020).

**e. Flavonoids**

Flavonoids are essential plant natural products that have polyphenolic moieties, but they are also EF derived. Due to their antioxidant, anti-inflammatory, antimicrobial, antimutagenic, and anticarcinogenic effects, they have significant miscellaneous biochemical, cosmetic, nutritional, and pharmaceutical applications for the treatment of various ailments, such as Alzheimer’s disease, cancer, and atherosclerosis. In addition, flavonoids are basic ingredients in the field of cosmetics (Panche et al., 2016).

**f. Saponins**

Saponins are a kind of glycoside compound in which the sugar moiety attaches to sapogenin through a glycosidic bond. Because of their antimicrobial properties, saponins play a defensive role in their symbiosis with host plants. Other applications are related to their anticancer, antinutritive, and anticholesterol properties (Nicoletti and Fiorentino, 2015).

**g. Phenols and phenolic acids**

Phenols and phenolic acids are a diversified class of SMs that are principally synthesized by plants and have also been isolated and identified from EF sources. Their major function is to act as signal factors in plant–microbial associations. They also act as defensive agents, promoting plant growth in nutrient-limited soil during symbiosis (Singh et al., 2011). This may be due to the higher concentrations of phenolic acids, phenols, and their derivatives in host plants inoculated with EFs (Mandal et al., 2010).

**h. Aliphatic and chlorinated metabolites**

Aliphatic and chlorinated metabolites have simple chemical structures but are considered xenobiotic compounds. These metabolites are widely biosynthesized by several forest and wood litter-degrading fungal species and endophytes. They have antibiotic activities against pathogenic microorganisms, insects, and algae, but they are also carcinogenic and genotoxic to animals and humans (Lin et al., 2011).

**i. Peptides**

Endophytic fungal peptides are another class of SMs that act as defense agents. They are protein forms with a molecular weight of less than 10 kDa (Ng, 2004). Significant scientific efforts have been directed toward identifying and isolating peptides as candidate drugs due to their high degree of interactions with their specific targets. Studies have reported endophyte isolates as potential sources of peptide-based drugs for the treatment of a variety of illnesses (Abdalla and Matasyoh, 2014). The most important group of anticarcinogenic and antifungal peptides, Leucinostatin, has been extracted from the fungal endophyte *Acremonium* sp., which is isolated from *T. baccata* (European yew plant) (Abdalla and Matasyoh, 2014).

**j. Steroids**

Steroids are natural chemical substances that are abundantly produced not only in plants and animals, but also in microbial communities. Steroids are bio-lipid-based terpenoids that have a structure of four fused carbon skeleton-based rings, which is characterized as the steroid nucleus or sterane. Steroids vary in structure and function because of the different oxidation states of functional groups attached to the rings (Theodorakidou et al., 2018).

It has already been demonstrated that endophytic steroids such as ergosterol exhibit extra pharmaceutical activities and natural roles in their producers. The 5a- and 8a-epidioxy ergosterols that have been isolated from *Nodulisporium* sp. have potent antimicrobial activities against a series of pathogenic microbial strains (Yu et al., 2010; Yang H. et al., 2015; Nowak et al., 2016).

## Substantial Challenges and Future Perspectives in Endophytic Fungi Studies and Conclusion

At present, endogenous biology is receiving increasing attention due to the great application potential of the chemicals secreted by EF–host symbiotic associations in sustainable agriculture and biomedicine. Scientists are interested in understanding the underlying mechanisms of endophytism and its biological and ecological roles. Its importance is highlighted by the large number of studies in the field of endophytic biological research (Hyde and Soytong, 2008; Deepika et al., 2016; Nützmann et al., 2018; Fang et al., 2019; Huang et al., 2019). Although EF research has attracted great attention, this field still faces substantial challenges to be addressed in the coming decades, including:

• The selection of suitable host plants and their healthy organs or tissues to identify and isolate new EFs and to dissect their related mutualistic or antagonistic signaling mechanisms during symbiosis (Strobel, 2018).
• The complexities in the process of artificial culture due to the aseptic or non-culturable characteristics of some fungal strains. It is important to introduce de novo bioengineering systems or to modify conventional isolation techniques to address this challenge (Stone et al., 2004; Zhang et al., 2006; Zhang P. et al., 2019).
• The biosynthesis of natural products of EFs, especially SMs, requires inducing stimuli from host plants or from symbiosis. In artificial axenic culture, EFs may not be able to synthesize the same chemicals as they do in a symbiotic continuum due to the absence of these plant-mediated stimuli or signals. For the EFs that have been successfully isolated and cultured, their SM production decreases with successive subcultures under axenic monoculture conditions (Cánovas et al., 2017). Therefore, it is also a challenge to monitor the *in vivo* stimuli of successfully isolated EFs under quasi-natural conditions and maintain their “competency” in culture.
• Fungal SMs play critical roles in understanding the endosymbiotic mechanisms between EFs and hosts. This endosymbiosis can be indirectly monitored by the transformation biogenesis of SMs under artificial culture conditions. Considering the significant impact of different types and concentrations of nutrients in artificial media in EF culture (Ruiz et al., 2010), this is not an easy job.

• The degradability of SMs extracted from target EFs needs to be addressed. Compounds isolated from a symbiotic continuum may be highly unstable *in vitro* or in axenic culture. Thus, it is difficult to obtain these required novel compounds in an artificial medium.
• The number of EFs that have been explored is limited. It is estimated that only 1-2% of about 300,000 plant species have been investigated, and at present, little information has been obtained from hydro-ecosystems, which means that the vast majority of EF symbiotic relationships remain elusive (Strobel, 2018).

In the current “omics” era, tools including genomics, epigenomics, transcriptomics, proteomics, and their related meta-omics (metagenomics, metatranscriptomics, and metaproteomics) (Zhang J. et al., 2019), will be extraordinarily supportive in illuminating the gray areas of myco-endophytisms and in tackling the aforementioned challenges to reveal complementary information on these symbionts and their interactions inside the internal niches of host plants (Peršoh, 2015; Nowack and Weber, 2018; Zhang J. et al., 2019). Furthermore, collaborative research between omics tools and other disciplines, such as combinatorial chemistry, will be more effective and fruitful in constructing novel molecular models of these EF–host interactions (Nowack and Weber, 2018).

## Conclusion

Secondary metabolites play a pivotal role in mediating biochemical communications between EFs and host plants. These biochemical communications guide the multidimensional interactions among EFs, host plants, and pathogens in their community and determine the host range of an EF and endophyte populations in a plant host. The biosynthesis of SMs during symbiosis is precisely regulated by several genetic mechanisms, including gene clustering, transcription factors, the altering of the genetic makeup of the host in the presence of EFs, and HGT. These regulation mechanisms may have coevolved with the initiation of EF–host symbiosis. Recently, SMs have attracted widespread research efforts due to their great biological potential in the discovery of modern medicines, sustainable agriculture, and industry. Further investigations at the molecular level may still be needed for gaining a better understanding the endophyte–host relationship in natural ecosystems at the genomic level, and for efficiently identifying the hidden genes involved in the biosynthesis of SMs and new compounds in axenic culture.

## Author Contributions

BA and WG: conceive the idea and write the initial draft of the manuscript. JL and QG: help organizing and editing the manuscript. QG and WG: perform the network construction and figure presentation. MK, JG, and SM: proofreading the manuscript. YY and WG: contribute to the final editing of the manuscript. All authors contribute in the interpretation of the manuscript and approve it.

## Conflict of Interest

The authors declare that the research was conducted in the absence of any commercial or financial relationships that could be construed as a potential conflict of interest.

## Publisher’s Note

All claims expressed in this article are solely those of the authors and do not necessarily represent those of their affiliated organizations, or those of the publisher, the editors and the reviewers. Any product that may be evaluated in this article, or claim that may be made by its manufacturer, is not guaranteed or endorsed by the publisher.
